# *Echinococcus granulosus *sensu lato control measures: a specific focus on vaccines for both definitive and intermediate hosts

**DOI:** 10.1186/s13071-024-06581-2

**Published:** 2024-12-23

**Authors:** Mehdi Borhani, Saeid Fathi, Majid Fasihi Harandi, Adriano Casulli, Jing Ding, Mingyuan Liu, Wenbao Zhang, Hao Wen

**Affiliations:** 1 State Key Laboratory for Zoonotic Diseases, Key Laboratory of Zoonosis Research, Ministry of Education, Institute of Zoonosis, College of Veterinary Medicine, Jilin University, Changchun, 130062 China; 2 State Key Laboratory of Pathogenesis, Prevention and Treatment of High Incidence Diseases in Central Asia, Xinjiang Medical University, Urumqi, China; 3 Department of Parasite Vaccine Research and Production, Razi Vaccine and Serum Research Institute, Karaj, Iran; 4 Research Center for Hydatid Disease in Iran, Kerman University of Medical Sciences, Kerman, Iran; 5 WHO Collaborating Centre for the Epidemiology, Detection and Control of Cystic and Alveolar Echinococcosis, Department of Infectious Diseases, Istituto Superiore di Sanità, Rome, Italy; 6 European Union Reference Laboratory for Parasites (EURLP), Department of Infectious Diseases, Istituto Superiore di Sanità, Rome, Italy

**Keywords:** Cystic echinococcosis, Epidemiology, Hydatid disease, Control measures, Vaccine

## Abstract

Echinococcosis, a neglected zoonotic disease caused by *Echinococcus* tapeworms, presents significant public health challenges worldwide. Cystic and alveolar echinococcosis has substantial health and economic impacts, necessitating effective prevention and control strategies. The present review provides a framework to expand our knowledge regarding key components of echinococcosis prevention and control, including phases, options, targets and available tools as well as current gaps and challenges in the field. Furthermore, we discuss the progress made in developing vaccines for the intermediate and definitive hosts and review the limitations and obstacles in vaccine development for definitive hosts. Abundant information is available on various aspects of the *Echinococcus* vaccine in sheep. Livestock vaccination effectively reduces *Echinococcus* transmission to sheep, offering a feasible control measure in intermediate hosts. However, vaccine development for the definitive host, i.e. dogs, exhibits significant challenges. Information gaps regarding the immune-mediated protective responses in dogs, repeatability of results, factors influencing the immune response, reinfection resistance, potential age-related decreases in worm burden and factors associated with the antifecundity effect are key challenges that should be addressed in canine vaccine development, and research collaboration, innovative technologies, and a deeper understanding of transmission dynamics are crucial. Multisectoral coordination under the One Health framework, with long-term political commitment and national and international cooperation, is critical for effective control in endemic areas.

**Graphical abstract:**

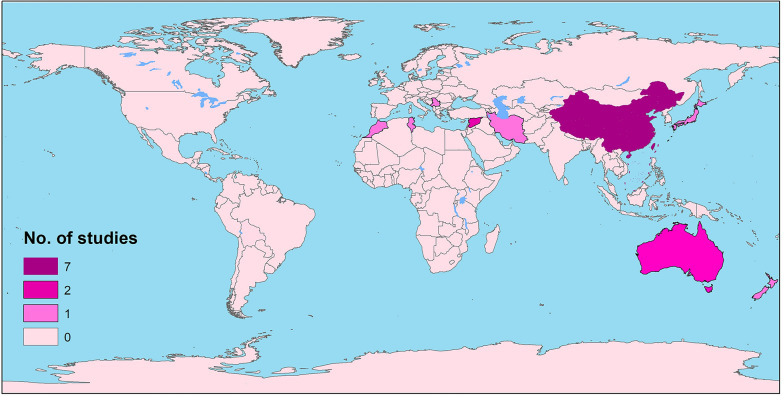

## Background

Echinococcoses are parasitic zoonotic diseases caused by several species of the genus *Echinococcus* that infect a broad spectrum of mammalian species, mainly canids, artiodactyls and perissodactyls, worldwide. Cystic echinococcosis (CE) and alveolar echinococcosis (AE) are the most relevant diseases from a global public health perspective; therefore, they are included among the neglected tropical diseases (NTDs). In particular, CE and AE are caused by *Echinococcus granulosus *sensu lato and *Echinococcus multilocularis*, respectively. CE in intermediate hosts (mainly livestock) and humans, as dead-end hosts, can be acquired by accidental ingestion of *E. granulosus* s.l. eggs excreted from the feces of definitive hosts such as wild and domestic carnivores (mainly dogs) [1].

Human CE is a chronic and disabling disease, with a large proportion of patients being asymptomatic for a long period, even throughout life, leading to an underestimation of the total number of infections. CE imposes considerable economic costs on low- and middle-income countries, including direct and indirect costs associated with human treatment costs and livestock-related losses [2, 3].

CE exhibits a highly variable human disease burden across various endemic regions, largely influenced by human behavioral risk factors as well as the diversity and ecological dynamics of animal hosts [4].

Dogs from semi-nomadic pastoral communities tend to exhibit higher infection rates due to the practice of home slaughtering, and subsequent increased accessibility to livestock offal. The risks are particularly pronounced in roaming and stray dogs, which have greater opportunities to scavenge infected carcasses. On the other hand, dogs that are kept as pets or guard dogs, with limited mobility and a diet primarily comprised of cooked food, typically show lower infection rates [5–8]. The proximity of stray dogs and un-dewormed owned dogs with humans and livestock can be drivers for CE transmission, thereby heightening the risk of human infection. Individuals most at risk include those directly engaged in livestock management, such as farmers and shepherds, rural populations, nomadic communities and veterinary workers. Furthermore, dog owners, particularly in rural or agricultural areas where dogs are not regularly dewormed, also face increased risk. These individuals may encounter *echinococcus* eggs present in the environment, thereby increasing their likelihood of exposure. Additionally, specific cultural and religious practices and socioeconomic factors can significantly heighten the risk of infection [4, 5, 9].

The cost associated with CE has been estimated at 0.01–0.04% of the national gross domestic product (GDP) in low- and middle-income countries, where 1 million people, at any one time, suffer from CE [10]. The World Health Organization (WHO) estimated an annual echinococcosis-related global burden of 19,300 deaths and approximately 871,000 disability-adjusted life-years (DALYs) (CE: 183,573 DALYs; AE: 687,823 DALYs). The overall annual cost of CE has been estimated at US$ 3 billion, including costs associated with both humans and livestock [11, 12].

All six WHO regions are affected by this disease. For example, the Eastern Mediterranean Region of the WHO (EMRO), with 22 countries, is a major hotspot of CE, with approximately 688 million individuals at risk of CE [13]. Moreover, 64,745 cases of human CE were identified in 40 European countries from 1997 to 2021 [14]; in this 25-year study, the mean annual incidence in Europe was 0.64 cases per 100,000 people [14]. Therefore, there is an urgent need for control interventions for CE in endemic areas.

Nevertheless, enacting the most effective strategy for the global control of CE will require an in-depth understanding of the benefits and costs of implementing control measures, such as human and livestock CE-related losses, and the factors that hinder interventions, such as sheep vaccination, to elucidate the technical difficulties in this regard [15].

Over the past decade, researchers have proposed a comprehensive control strategy for CE, which includes treating dogs with praziquantel (PZQ), vaccinating sheep with EG95, combining both strategies, enhancing slaughterhouse infrastructure, regulating home slaughter practices and promoting health education initiatives [9].

Although several interventions are available for control measures, no canine vaccine has been developed. Livestock vaccination can provide an adjunct to improved CE control, which is highly effective in intermediate hosts, and vaccine development for targeting the definitive dog host can potentially reduce the parasite biomass within the dog intestine in endemic areas [16].

This review offers a framework for expanding our knowledge about the prevention and control of echinococcosis. We also discuss the progress in developing vaccines for the definitive hosts and provide an overview of the limitations and obstacles encountered in vaccine development for dogs.

### Options, phases, targets and tools needed for the control of *E. granulosus* s.l.

There is a need for large-scale health policy interventions for the control of many infectious diseases, particularly NTDs such as echinococcoses, for which the implementation of control measures is highly important. Successful cases in places like New Zealand and the Falkland Islands demonstrate that it is possible to transform highly endemic areas into those with sporadic cases and potentially eliminate the disease [9]. However, achieving thorough control of echinococcosis poses significant challenges, especially in continental regions compared to island areas. Moreover, control programs face significant challenges in underdeveloped regions with nomadic lifestyles due to factors like remote locations, poor infrastructure, unengaged populations and the inherent mobility of nomadic populations.

Although some successes have been observed in specific communities, a more effective strategy may be a ‘One Health’ approach, integrating multiple zoonotics for efficient resource allocation and outreach. Such collaboration can improve the overall effectiveness of disease control efforts in these areas [17–22]. Thus, given that a one-size-fits-all strategy fails to achieve success, CE control options have been recommended over four phases, including the planning, attack, consolidation and maintenance [22].

Policymakers in endemic areas need to enact control programs. Decision-making planning (phase 1) needs to explore prerequisites before the start of the attack phase (intervention/control measures), including cost-benefit analysis, cost-effectiveness analysis, burden of disease, presence of a surveillance system, registration system for humans, households and dogs, high-quality baseline data with different approaches for intermediate hosts (livestock), humans and definitive hosts (dogs), identification of intervention target areas, selection of options, long-term funding and support of relevant authorities for a certain period, choosing staff for control programs, science communication, treatment and follow-up support for humans and implementation of pilot schemes [9] under the terms of One Health, which need to be employed by endemic regions and their countries via practical planning. However, each region has its unique conditions. Understanding the acceptability of interventions among various stakeholders, including breeders, local authorities and health providers, is essential for the success of control programs. This understanding not only enhances community engagement but also allows for the tailoring of interventions to maximize their effectiveness [23]. Cost-effectiveness analysis is essential for evaluating health interventions, particularly in addressing the overall cost associated with various programs.; however, it faces limitations in using DALYs as a Health Adjusted Life Years (HALYs) metric that do not adequately capture the comprehensive health and financial impacts of costs associated with animal health [24, 25]; thus, economic losses due to disease in animals are usually estimated in monetary terms (monetary losses associated with humans and livestock; direct and indirect losses) [26]. DALY application in cost-effectiveness analyses, particularly through measures like cost per DALY averted, provides a crucial framework for decision-makers to optimize resource allocation in public health [27–30]. Although alternative metrics including zoonotic DALYs (zDALYs) are proposed to estimates the impact of a zoonotic disease to animal and human health [24], they are still underutilized in echinococcosis studies [25].

Economic evaluations are complicated by unclear cost breakdowns and inconsistencies in documenting time and overall program delivery costs, such as those for dog deworming, bait delivery, sheep vaccination, materials for drug administration and cold chain storage as well as costs for transportation, educational campaigns, equipment, surveillance, monitoring, diagnostic tests, tools and staff wages, etc. [25, 31–34]. Many studies often overlooked expenses related to geographic factors affecting access such as remote areas, underscoring the need for thorough cost documentation [25, 35, 36].

Furthermore, exploring integrated control strategies for CE alongside other NTDs and zoonotic diseases can be recommended, as such approaches have the potential to enhance coverage and reduce costs, especially in impoverished communities [25, 35, 37], thereby highlighting the necessity for further investigation into the cost-effectiveness of integrated control programs.

Since the 1960s, echinococcosis control programs have been employed worldwide, with the outcomes being considerably effective, eventually effective, or less effective [9]. In the attack phase, three potential options could be evaluated: (i) treating dogs with PZQ on a monthly to quarterly schedule based on local disease burden (reinfection rate), (ii) vaccinating livestock and (iii) combining both approaches.

An effective integrated control program that combines a suitable regimen of PZQ dosing (administered 4 to 8 times annually) with EG95 vaccination for livestock, alongside the control of free-roaming dog populations when needed, is expected to decrease or eliminate CE transmission in various endemic areas. In addition, it is advisable to adopt complementary strategies, such as: (i) enhancing slaughterhouse infrastructure, (ii) regulating home slaughter practices and (iii) promoting health education initiatives. Such strategies are supported by effective control programs in island-based initiatives (Iceland, New Zealand, Tasmania and southern Cyprus) and in a few effective continental schemes (such as those in Chile, Argentina and Uruguay) and indicated in other recommendations [9, 22, 35, 38–40]. The success of island control programs is influenced by the unique context of each island, particularly concerning the relatively manageable target populations [22]. Although substantial progress has been made in reducing parasite prevalence in animal populations and decreasing disease transmission to humans in continental areas, such as the Rio Negro Province in Argentina, it is important to recognize that the intricate characteristics of these areas differ markedly from those present in island environments.

On the other hand, control program failures were caused by poor stray dog management, dependence on owners for treatment, limited funding, insufficient baseline data, a shortage of qualified staff and premature funding termination [9]. Logistical and financial obstacles, along with administrative constraints and political instability, impede the effectiveness of interventions in endemic countries [35, 40, 41]. Addressing these issues requires multi-sectoral coordination and a comprehensive One Health approach that integrates the expertise and resources of both veterinary and human medicine [41]. Tools for enhancing intervention planning include PZQ for dogs, coproantigen ELISA tests for dogs, the EG95 vaccine for sheep, portable ultrasound for human screening in endemic areas, computer-based models for cost-benefit analyses of different scenarios of control programs and predictive combinations of interventions. A pivotal element in the control of echinococcosis involves effective surveillance to track the incidence and prevalence of CE in humans and livestock through age-specific surveillance data, as well as the prevalence of echinococcosis in dogs, while estimating DALY and the monetary burden of the disease. The absence of a solid foundation of baseline data and consistent follow-up assessments impede the evaluation of control measures for echinococcosis. This deficiency not only hinders the effective measurement of intervention success but also complicates the justification for the ongoing expenditure necessary to sustain these resource-intensive strategies [3, 9, 35, 42]. Although there is a wealth of information on human and animal echinococcosis from various endemic regions, age-specific data are often lacking in many studies [3], especially in low-income or developing countries, including Central Asia [43]. This gap highlights the need for more comprehensive studies to better understand the dynamics of the disease across different age groups [3].

The consolidation phase of echinococcosis control programs involves transitioning from the initial attack phase to ongoing surveillance of infected livestock, quarantining infected properties, encouraging dog owners to use PZQ, and incorporating the EG95 vaccine into routine vaccination schedules, all of which must be maintained permanently for effective disease management. The maintenance phase is a permanent stage that follows control activities, emphasizing ongoing vigilance through meat inspections and hospital records, and incorporating trace-back procedures, as well as movement controls for dogs to support disease elimination efforts [9].

The vaccine successfully prevents new infections, but it does not remove existing cysts. This highlights the necessity for continuous vaccination until all grazing animals are replaced. The successful management of this disease necessitates a multidisciplinary collaboration rooted in the One Health framework. It requires addressing real-world barriers, reporting costs (cost–benefit analyses for expandability and sustainability), sustained government commitment, substantial financial investment, fostering community engagement and strengthening stakeholder involvement to ensure sustainability and long-term disease management [44].

While vaccination is a crucial step, the process of eliminating the disease may extend over a decade. Therefore, sustaining health education and deworming initiatives post-vaccination will be essential to ensure long-term success in combating the disease [44]. Moreover, long-term programs are vital for effectively managing CE as a chronic endemic disease, despite ongoing challenges.

### Targeting CE in livestock

#### Slaughterhouse inspection and livestock management

Proper oversight by the veterinary sector is essential in slaughterhouse regulation. However, some municipal slaughterhouses may lack the necessary infrastructure to effectively dispose of condemned organs, resulting in the availability of offal for dogs. Hence, there is an urgent need to enhance the inspection process of carcasses and improve slaughter facilities overall as these are crucial aspects in controlling and combating echinococcosis. In addition, addressing illegal slaughtering, as well as the cultural and religious practice of home slaughter during specific events, is paramount. Moreover, addressing the lack of awareness of the disease among animal owners is crucial, as these factors are critical in combating the transmission of CE [3, 45].

Changing centuries-old habits in home slaughter requires deeply rooted cultural, socioeconomic, and religious factors, including social events, nomadism and traditional animal husbandry along with individual attitudes and inadequate infrastructure for standard industrial abattoirs [46–50], which can be quite challenging. While it is feasible to modify these longstanding habits through legislation, educational initiatives aimed at reshaping public attitudes and the establishment of standard industrial abattoirs accompanied by sustainable veterinary supervision and alternative practices that harmonize traditional slaughter methods with existing regulations [46], significant economic and social development often play critical roles in facilitating such transitions.

Cultural traditions play an important part in shaping societal norms and values, and even when economic conditions and infrastructures are improving, resistance to new change can continue to exist when such traditions carry important cultural or spiritual significance. Consequently, any approach to effect a shift in such practices would need to address the linked issues of cultural identity, religious conviction and economic factors and allow communities a say in dialogue and understanding, at the same time offering more sustainable alternatives that are humane and fully regard their tradition.

The current body of literature inadequately explores the nature and extent of home slaughter practices across different regions, revealing a significant knowledge gap in our understanding of the epidemiology of zoonotic diseases related to this practice. Addressing this gap can lead to the development of informed public health initiatives and policy interventions necessary for formulating effective health strategies, which enhance food safety and reduce the global transmission of zoonotic diseases, particularly in low- and middle-income settings.

#### Livestock vaccination

Regarding control via the vaccination of animal intermediate hosts, the EG95 anti-oncosphere vaccine for *E. granulosus* was first described by Lightowlers et al. in 1996.

The EG95 vaccine has been extensively demonstrated effective in reducing *E. granulosus* transmission to sheep (95–99% protection) in Argentina, Australia, China and New Zealand [51–59]. Studies in multiple countries, such as Australia, Argentina and China, have demonstrated that the recombinant EG95 vaccine reliably provides substantial protection (ranging from 95 to 99%) to sheep against *E. granulosus* egg infection challenge [52, 60]. The vaccine is now on the market in Argentina and China, where it is being used in national programs to control disease and lower rates of human infection by reducing parasite transmission [61]. It has not yet been scheduled in integrated control programs in many countries in which CE is endemic, especially WHO EMRO regions. The use of the EG95 vaccine to immunize intermediate hosts has been suggested as a pillar of control to effectively decrease the number of cysts accessible to definitive hosts, serving as an additional control measure [58].

The analyses of short-read data from various countries, including Australia, Argentina and China, have indicated excellent amino acid conservation of the EG95-1 vaccine. This conservation is observed across different host species, such as sheep, cattle, buffalo, goats, pigs, dogs/dingos and even humans, all of which are affected by *E. granulosus* (G1 or G3) infection [62]. However, the EG95-1 vaccine has antigenic variation compared with *E. canadensis* G6/7, encouraging further investigation given the conceivable effect on vaccine antigenicity/efficacy [63–65].

Anecdotal evidence suggests that sheep vaccination requires relatively little intervention, typically consisting of two full-dose vaccinations annually. In contrast, PZQ administration requires a higher frequency of dog dosing, often as frequently as eight times per year [66].

Truncating the original EG95 cDNA to remove short hydrophobic stretches from both the amino- and carboxy-terminal regions of the protein (EG95NC^−^) improved the expression of the soluble component of the *Escherichia coli*-expressed antigen without compromising its ability to provide protection [67]. As a new generation vaccine, EG95NC^−^ bacterin was shown to induce 98% protection against CE in a sheep vaccine trial, streamlining production and revealing new strategies for producing the vaccine and improving vaccination uptake [68].

As documented by field trials, the EG95 vaccine provides long-term immunity for at least 5 years after the third vaccination (with a suggested dosing scheme of two subcutaneous immunizations in recently weaned animals followed by a single booster immunization when sheep are approximately 1 year of age) [56, 58, 69], providing a sufficient duration of protection against infection or fertile cyst development. In cattle, two injections induce protective immunity that may last for 12 months [53, 70].

Experimental trials conducted across various countries, including New Zealand, Australia, China, Argentina, Chile and Romania, have convincingly demonstrated the reliability and effectiveness of the EG95 vaccine [22, 53, 59, 71–73].

The CE control program, launched in 1980 in Rio Negro Province, has effectively decreased the prevalence of *E. granulosus* in both dogs and humans. This success can be attributed to the implementation of regular PZQ treatments, vaccination efforts, ongoing surveillance and community education initiatives. By 1997, the program had achieved a remarkable reduction of 90% in infected dogs and 95% in humans, and by 2015, there was a 65% decrease in infections among sheep [57, 74]. However, the remaining infected sheep continue to pose a risk of causing persistent CE cases, especially among children [57], while program pressure for maintenance resulted in a continuous decrease in prevalence in children [75].

Preliminary data of a field trial in the remote Rio Negro Province of Argentina highlighted a significant reduction in *E. granulosus* infection rates among vaccinated lambs during the 3 years beginning in December 2009, which prevented infection in animals up to 3 years old [57]. A second study of the trial showed vaccination could improve the effectiveness of CE control activities, even in a remote environment where it is difficult to deliver the program and only approximately half the lambs born in the communities are fully vaccinated [76]. Additionally, a sustained increase in EG95 antibody levels in the sheep population following the third immunization was achieved in Rio Negro, revealing a serological pattern required for higher protection [56]. To illustrate the effect of sheep vaccination on the transmission of CE to dogs and, consequently, its potential impact on human transmission, an 8-year study conducted in Rio Negro revealed a significant reduction in the prevalence of ovine CE in both the number of cysts and the number of farms with infected sheep. Furthermore, the proportion between the positive samples during the baseline in 2011 and the follow-up status in 2021 was statistically significant according to the Copro-ELISA test. Stable rates of infection in dogs were significantly lower than at the beginning of the program, with a remarkable reduction in transmission to humans [58].

The prevalence among humans in the region dropped significantly from 5.6% in 1984 to just 0.1% in 2016. The ongoing control program has consistently targeted the parasite over several years, resulting in a steady decline in prevalence among children, even though complete eradication has not been achieved [75].

Insights from 12 years of study in Río Negro have provided valuable evidence regarding the long-term effectiveness of the EG95 vaccination program against CE. Since its implementation in 2009, the program has successfully reduced CE prevalence among both sheep and goats following the administration of three vaccine doses. The combination of the vaccination program with PZQ (4 times/year in vaccinated and control areas, plus 2 additional deworming in 2018), has proven to be cost-effective, significantly impacting prevalence across various hosts and providing potential for maintenance of the vaccine over time to offer sustainable long-term financing [73]. Despite receiving PZQ treatment every 3 months, dogs continue to be infected and move between vaccinated and control areas [44, 57], indicating the importance of dog roaming control in areas where programs are conducted to support disease elimination efforts. The control strategy of CE in Río Negro and Chile serves as a prime example of the One Health approach, which addresses the challenges posed by *E. granulosus* s.l. However, aligning control strategies with socio-cultural factors, especially in indigenous communities, is crucial for success. Community engagement and sustained efforts in dog deworming and routine vaccination of grazing animals are essential, alongside a robust surveillance system, to achieve lasting control of this public health concern [44]. Additionally, applications of the EG95 vaccine since 2020 in the Aysén Region in Chile demonstrate its efficacy in reducing CE prevalence in vaccinated sheep along with quarterly dosing (deworming) after 3 years of administration. This supports the integration of the EG95 vaccine into comprehensive control programs, underscoring its role as a crucial component in managing echinococcosis [44].

Since 2010, a comprehensive control program in the Campania region of southern Italy has combined multiple strategies, including surveillance, diagnosis, targeted treatment, and public outreach. These efforts have led to a notable decrease in parasite infection rates in livestock, with reductions of up to 30% in sheep observed over 8 years. This successful multi-disciplinary strategy underscores the importance of a One Health approach in developing CE control programs, which could be applied to other endemic Mediterranean regions [77].

Mathematical modeling of various *E. granulosus* control options [42] suggests that a combination of vaccination with EG95 and biannual treatment of 80% of dogs with PZQ would allow the length of control programs to be reduced, achieving a fast and robust reduction in CE incidence in sheep. Field evaluation of sheep vaccinated with EG95 for CE control in the Middle Atlas Mountains in Morocco, North Africa, revealed that the EG95 vaccine effectively decreased *E. granulosus* transmission to sheep, whereas treatment of owned dogs with PZQ alone every 4 months was insufficient and demonstrated no synergy with the vaccination [60]. The EG95 vaccine findings from Morocco further suggest its promising applications in other regions. Dogs need to receive frequent treatment with PZQ for a considerable duration (several decades) since they are prone to rapid reinfection [78].

Over the past of 19 years in China, the national incidence rate of echinococcosis demonstrated that the adoption of control measures has led to a reduction in infection rates of nearly 50% from 2004 to 2022, decreasing from 0.3975 to 0.1944 cases per 100,000 person-years. In addition, following the implementation of sheep vaccination with the EG95 vaccine in 2016 in the seven provinces and regions in northwestern China where echinococcosis is endemic, national incidence has continued to decline every year since 2017. However, segregated data on changes in the incidence of AE and CE are currently unavailable. The trends observed in echinococcosis, as well as the epidemiological patterns of CE and AE, align closely with the timelines of dog deworming initiatives that began in 2005 and the introduction of EG95 vaccination for livestock in 2016, highlighting the effectiveness of targeted control strategies [72].

### Targeting the definitive host (dogs)

#### PZQ dosing, arecoline purgation and dog population management

Focusing on definitive hosts, particularly dogs, can be considered the first-line approach for implementing control strategies. Three options, namely, PZQ dosing, arecoline purgation and dog population management (catch, neuter and release [CNR] and culling), have been introduced for targeting dogs [9, 79–81].

PZQ dosing for dogs is crucial for controlling CE in endemic areas. Understanding reinfection rates [41] and exploring slow-release formulations of PZQ are steps toward improving mass treatment for dogs and overall efficacy [82, 83].

As dogs can become reinfected after treatment, the reinfection rate serves as an indicator of CE transmission intensity in endemic areas. This rate influences the frequency of dog deworming, making it vital for planning effective CE control programs [41]. Explaining the differential effect of the reinfections on a given infection pressure, and/or no reinfections, which concerns the observed reduced prevalence among the definitive host, in more detail is still required [84].

Theoretically at least, continuous dog dosing can significantly reduce the transmission of CE. Indeed, there is evidence for using PZQ in continental areas with different application frequencies, especially in terms of reducing transmission to humans. However, the practical implementation of these programs faces challenges. Achieving low transmission levels typically requires over a decade of intensive interventions that rely on frequent dog treatment (treatment adherence).

Continuous dog dosing campaigns require enhancements in coverage and the establishment of sustainable resources and infrastructure (governmental support and logistical enforcement) in endemic areas, which are often the most economically disadvantaged [57, 81, 85]. Sustaining these initiatives within nomadic and semi-nomadic pastoral communities presents significant logistical challenges [55, 86, 87].

While innovative technologies such as geospatial technology (GPS data loggers), Internet of Things (IoT) Remote Monitoring Systems and praziquantel-laced baits delivered by unmanned aerial vehicles (UAVs) [33, 36, 77, 88] offer promising opportunities for improving coverage and efficiency, the high logistical costs associated with sustained PZQ-based control programs remain a major barrier. The disparities in cost-sharing models complicate the implementation of effective treatment regimens. For instance, dog owners may face substantial out-of-pocket expenses that deter them from adhering to treatment protocols. In contrast, the provision of PZQ to dog owners at no cost serves as a vital incentive; however, without comprehensive support to address other associated costs, intervention compliance may remain suboptimal. A nuanced understanding of the various cost-sharing approaches is crucial for the successful implementation of control programs. Policymakers must prioritize government funding and develop supportive frameworks that empower dog owners, ultimately leading to better outcomes.

Differences in the pricing of PZQ reflect historical factors, including the transition from patented to generic formulations after 1994, along with factors such as shipping costs and purchasing volume (bulk purchases), which have led to inconsistencies in the reported costs. Additionally, uncertainties surrounding the expenses associated with program delivery, such as the frequency of mass drug administration, further obscure accurate cost assessments. Improved data clarity is necessary for effective economic evaluations and informed policy-making, as many details are not completely documented in the studies [25].

Reinfection studies play a crucial role in understanding the epidemiology of echinococcosis in dogs. They help address key questions and provide essential baseline data (i.e. prior to a planning phase) necessary for effective CE control and prevention strategies [41]. Various studies indicate that dogs should be dosed with PZQ at least every 45 days (four times a year), aligning with WHO recommendations [20, 38, 89–91].

Understanding dog reinfection dynamics in endemic areas is essential for dog deworming based on regional endemicity. The variation in reinfection patterns across different regions underscores the importance of further investigation. The gap in comprehensive data on reinfection rates in many endemic locations poses challenges to developing informed and effective strategies. To determine an effective dog deworming frequency in certain endemic areas, it is essential to balance efficacy with operational sustainability and feasibility, necessitating field and modeling studies for optimal dosing frequency for maximum impact on CE transmission [41].

Currently, a preventative vaccine for canine echinococcosis is unavailable. Nevertheless, the implementation of a comprehensive immunization program designed to target both definitive and intermediate hosts may hold great promise as a pivotal and imperative strategy for echinococcosis control, management, and prevention.

#### Vaccination of definitive hosts

Although livestock vaccination, which is highly effective in these intermediate hosts, can provide an adjunct to improve the control of CE; vaccine development targeting the definitive host, dogs, can also be considered for the suppression or reduction of egg development [35, 92, 93]. However, no vaccine is available, and significant questions remain because of incomplete knowledge regarding dog immunity and vaccination.

#### Canine vaccine candidates

Concerning infection-acquired immunity in canids, studies suggest a partial decline in the susceptibility to reinfection and age resistance, which leads to decreased *Echinococcus* worm burdens in naturally infected canines [94–102]. These findings likely resulted in some level of protective immunity following a natural infection [103], which explained in detail below. However, such findings need to be confirmed in well-designed studies with empirical evidence [16, 92, 104].

The available findings do not provide definitive support for the existence of immunologically mediated protective responses against *E. granulosus*.

Notably, the current findings do not definitively support the occurrence of immunologically mediated protective responses against *E. granulosus*. The interpretation of the results may be a matter of debate [9, 16], and the repeatability of these findings requires further evaluation.

**Fig. 1:**
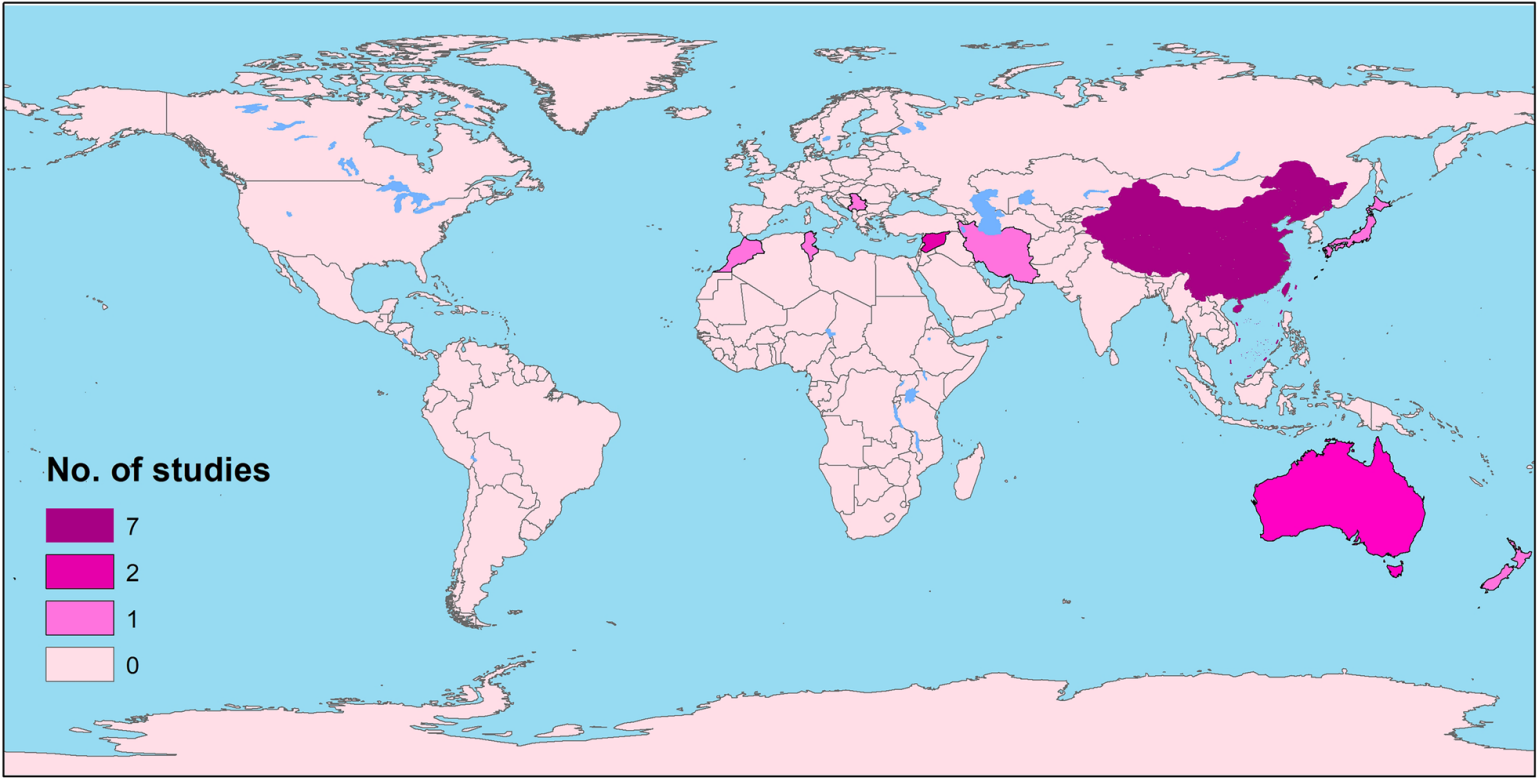
Mapping the global distribution of *E. granulosus* canine vaccination studies

To date, several vaccine-candidate antigens have been employed in different regions (Fig. 1) to immunize definitive hosts against *Echinococcus* infection, such as crude antigens (hydatid cyst antigens and protoscolex antigens), adult tapeworm tissue, secretory antigens derived from in vitro cultures of adult *E. granulosus* tapeworms and recombinant protein-based vaccines (Table 1).

**Table 1:** Experimental assessments conducted for dog vaccine

Area	Period	Surveillance	Intervention	Antigen	Reported results	References
Syria	1933	Autopsy	Challenge fresh fertile hydatid cysts	Scoleces and the germinative membrane of *Echinococcus granulosus*	Significant reduction of *E. granulosus* in the vaccinated group	[105]
Syria	1936	Autopsies	Challenged with 3000–50,000 PSCs	Scolex antigen, scoleces and membranes antigen, sterile cysts antigen, new scolex antigen	A marked decrease in the incidence and degree of intestinal infection with *E. granulosus* as compared to the controls	[106]
New Zealand	1962	Autopsy	Challenged with 50,000 PSCs	Freeze-dried scoleces	Lowest average worm burden	[107]
Yugoslavia	1968	Autopsy	Challenged with 3000–5000 PSCs	Larvae of *E. granulosus*	A significant proportion of protection was observed in dogs	[137]
Australia	1975	Autopsy	Challenged with about 80,000 PSCs	Worm secretory antigens	A significant decrease in egg production, and remarkable reduction in the number of proglottides per worm	[109]
Australia	1977	Autopsy	Challenged with 60,000 and 50,000 PSCs	Secretory antigen of adult *E. granulosus*	Mean proglottid numbers of l.l1.9 and 100% inhibition of egg production	[110]
China	2006	ELISA, and autopsy	Challenged with 480,000 PSCs	Recombinant proteins egM4, egM9 and egM123	Induced a very high level of protection in terms of suppression of worm growth	[78]
Morocco and Tunisia	2007	ELISA, and autopsy	Challenged with 75,000 PSCs	Recombinant proteins EgA31 and EgTr	Vaccinated dogs showed reduced parasite burden and slower worm development rate in all remaining worms, but low IgA and IgE responses with no differences from controls	[113]
China	2010	ELISA, and autopsy	Challenged with 200,000 PSCs	Eg M9	Protection rate of vaccination group reached 86%	[112]
Japan	2013	ELISA, and autopsy	Challenged with 500,000 scoleces. multilocularis protoscoleces	Surface glycoprotein from* E. multilocularis*	87.6% reduction in worm numbers compared to control dogs, IgG levels gradually increased	[138]
China	2018	ELISA, and autopsy	Challenge with 200 000 PSCs	Recombinant proteins EgM9 and EgM123	Induced significant protective efficacy in terms of worm burden reduction and suppression of worm growth and egg production	[124]
China	2018	ELISA, and autopsy	Challenged orally with 250,000 PSCs	Eg M9 and Eg M123	IFN-γ and IL-10 in the sera showed an elevated levels, specific Ig G antibody was locally detected in the mesenteric lymph nodes and small intestine	[136]
China	2021	ELISA, and autopsy	Challenged with 100,000 PSCs	rEgHCDH	87.2% reduction in the number of *E. granulosus* worms, inhibition rate of development and maturation of worms was 67.7%	[115]
China	2021	ELISA, and autopsy	Challenged with 100,000 PSCs	Tetraspanin TSP11	76.80% reduction in the number of worms	[114]
Iran	2021	ELISA, and autopsy	Challenged with 105,000 PSCs	Multi- epitopes rEGVac (EG95, Eg14-3-3 and EgEnolase)	The immune protection efficacy against E. granulosus was notably significant in dogs, whereas its efficacy in sheep was comparatively lower	[116]
China	2023	ELISA, and autopsy	Challenged with 70,000 PSCs	rEgTIM, rEgANXB3, rEgADK1, rEgEPC1, rEgFABP, and rEgA31	Significant protectiveness, with a worm reduction rate, the level of IgG in the vaccinated dogs was significantly higher than that of the control dogs	[117]

Numerous studies have developed vaccines against the growth of *E. granulosus* and infection in dogs. In 1933, Turner et al. reported a reduction in the number of *E. granulosus* adult tapeworms following immunization with antigens derived from protoscoleces and the germinal layer of hydatid cysts [105]. Turner et al. (1936) later reported partial resistance to *E. granulosus* infection in dogs vaccinated with hydatid cyst antigens [106].

In 1962, Gemmell reported less worm development in dogs vaccinated with freeze-dried preparations of either adult tapeworm tissue or scolices than in unvaccinated dogs [107].

In 1970, 16 dogs were challenged orally with single or double doses of *E. granulosus* protoscoleces irradiated at 25 kR and killed 58 days post-challenge, where examination revealed that the vaccinated dogs acquired robust resistance to infection following immunization [108].

Herd et al. (1975) demonstrated that vaccination was associated with arrested development of the worms and a significant reduction in the proportion of mature worms containing eggs among a group of six mature worm-free dogs immunized with secretory antigens derived from in vitro culture of adult *E. granulosus* tapeworms compared with that among five control animals that received adjuvant alone [109]. However, an identical immunization schedule involving two groups of 10-week-old Labrador crossbred pups was not capable of producing the same vaccine-associated effect, as considerable resistance to *E. granulosus* (i.e. decreased worm length, arrested development and egg production inhibition) was observed in two immunized dogs and three control dogs. However, a strong immune response has been reported in immunized dogs that were resistant, whereas control dogs exhibited no immune response [110]. A reduction in fertility, rather than a direct immune effect on germinal development, has been suggested to be associated with arrested development [110].

In 1980, Aminzhanov described the findings of a trial involving two groups of dogs: dogs immunized with irradiated antigenic extracts of hydatid fluid, protoscoleces and the germinal or cuticular membrane of adult *E. granulosus* once or twice. A greater reduction in burden and prolonged development were observed with the use of protoscoleces irradiated at 30,000 or 60,000 R [111].

In an experiment, Gemmell et al. (1986) assessed the effects of repeated infections on the number, size and fecundity of the worms in 16 dogs; wide variation was found in susceptibility (worm numbers) from one infection to the next, and they hypothesized there was a density-dependent effect, as 99.9% of the dogs in the experiment were predicted to become resistant to heavy worm burdens by the 12th challenge. However, numerous animals exhibited no decline in worm burden or development during repeated infections, whereas a decrease in the size and fecundity of *E. granulosus* was observed in some dogs after repeated challenge infections, and retarded growth of the whole worm population was found in some experimentally infected naive dogs [101]. Nonetheless, a clear indication of immunity was not observed in any of the animals [9].

Vaccination with soluble native proteins isolated from *E. granulosus* protoscoleces was linked to the suppression of worm growth and egg production among immunized dogs [78]. Compared with PBS and GST, recombinant EgM4, EgM9 and EgM123 (3 doses of 80 µg of protein) were evaluated for their ability to induce immunity in dogs against *E. granulosus* challenge, and the three recombinant antigens were reported to suppress worm growth, egg development and embryogenesis, with a very high level of protection (97%–100%) observed [78].

A second vaccine trial by Zhang et al. (2018) demonstrated that EgM9-GST and EgM123-GST mixed with Quil A were capable of inducing similar protective efficacy in dogs, effectively reducing worm growth and suppressing egg production, as shown with the proteins emulsified with Freund’s adjuvants in their previous trial.

Moreover, a separate study was performed to evaluate the protective efficacy of rEgM9 vaccination in dogs, and the vaccine induced a remarkable antibody response to inhibit parasite development [112].

A live attenuated *Salmonella* mutant strain carrying a vector expressing tropomyosin (EgTrp) and a fibrillar protein similar to paramyosin (EgA31 (from an adult *E. granulosus* worm) was employed to immunize two groups of five dogs compared with groups of controls (*Salmonella* vector control [3 dogs] and no treatment control [3 dogs]) in a pilot study in Morocco and Tunisia. The study reported a decreased adult worm burden in vaccinated animals (70% to 80%), whereas vaccinated dogs exhibited a protective effect [113].

A recombinant antigen of tetraspanin 11 (rEg-TSP11) from *E. granulosus* mixed with the saponin adjuvant Quil A was employed to vaccinate a group of three Beagles in contrast to Quil A only (3 Beagles) and the control treatment (PBS; 3 Beagles), resulting in a significant reduction in the worm burden and prevention of segment development in the dog model [114].

A recombinant 3-hydroxyacyl-CoA dehydrogenase (rEgHCDH) vaccine was reported to be capable of inducing partial immune protection against *E. granulosus* infection in Beagle dogs (3 animals), which presented a decreased worm burden and inhibited worm development, as well as induction of the production of specific antibodies, in contrast with the PBS control group (3 Beagle dogs) [115].

Immunization with a multiepitope recombinant vaccine including the three putative vaccine antigens EG95, Eg14-3-3 and EgEnolase was administered to five segregated groups (*n* = 3 per group, consisting of local stray puppies aged 5–9 weeks). The vaccination protocol employed two subcutaneous doses administered 28 days apart. Notably, this intervention was capable of inducing immunoprotection against *E. granulosus*, as observed by the absence of any discernible infection burden within the intestines of vaccinated dogs through microscopic examination. An identical outcome was reported in the histopathological analysis in contrast to the canines within the positive control group. Nevertheless, the study has some limitations, most notably the limited number of animals per group [116].

A recent study reported the efficacy of protein vaccine components, namely, rEgTIM&rEgANXB3, rEgADK1&rEgEPC1 and rEgFABP-EgA31, in protecting against *E. granulosus* via the evaluation of three groups of six Beagle dogs with worm burden reduction rates of 71%, 57% and 67%, respectively. Furthermore, the vaccinated subjects exhibited considerable suppression of worm growth, as verified through a discernible reduction in body length and width. A particularly remarkable discovery emerged from the coadministration of rEgTIM&rEgANXB3 vaccine to Beagle dogs, which employed a two-injection schedule, which exemplified exceptional stability as demonstrated by the minimal standard deviation, with a notable 71% reduction in worm burden [117].

Although evidence from a number of studies hints at the existence of a potentially protective immune response against reinfection in canids following natural infection, it is imperative to validate and substantiate such findings through well-designed empirical studies.

### Research gaps in definitive host vaccination

#### Vaccination efficacy, challenges and shortcomings of studies

Following the introduction of a potential candidate vaccine, empirical investigations that characterize infection dynamics in naturally infected dog populations and various aspects of canid immunity against *Echinococcus* after natural infection and vaccination are imperative. Studies are crucial in establishing conclusive evidence regarding canine immunity [92]. First, well-designed trials with optimal protocols and adequate sample sizes are particularly important for extrapolating result. To ensure congruity, it is important to consider the characteristics of the experimental dogs, the uniformity of the infection course among the groups and the uniformity of breed or type and age within the study design, underscoring the need for more robust methodologies. Addressing these factors will help validate preliminary observations and deepen our understanding of this complex area. However, the unique challenges and difficulties associated with conducting experiments with dogs cannot be overlooked.

Additionally, such studies help to confirm the presence of immunologically protective immune responses in canids following exposure to the pathogen in its natural state [103]. Furthermore, these studies should provide insights into the factors that influence immune response results, such as the required worm burden for conferring protection, the proportion of infection-acquired immunity and the proportion of maturing worms under varying levels of naturally acquired immunity as well as the protection of infection-naive or previously infected dogs [9, 92] and age-associated resistance. Additionally, it is essential to consider that the antifecundity effects observed in vaccinated dogs could stem from precocious rather than retarded development. Factors influencing the antifecundity effect of vaccination should be examined, which may involve evaluating dog feces before necropsy to ascertain the occurrence or exclusion of precocious worm development as well as the potential progression of relatively slow-developing worms into fully gravid worms over subsequent days [9, 118].

Despite previous findings suggesting lower *E. granulosus* burden or prevalence in aged animals, which indicated resistance to reinfection and a potentially age-related decline in *Echinococcus* worm burden [98–100, 102, 119], it is crucial to explore whether the likelihood of reinfection is lower in older, naturally infected canines than in their younger counterparts.

In the subsequent phase, upon substantiating the efficacy of protection, determining the longevity of vaccine protection in dogs becomes imperative [35]. Specifically, an assessment of changes in immunity and the duration of protection in vaccinated animals, as well as an exploration of the underlying immunological mechanisms that govern the persistence of protection, is needed to accurately determine the longevity of vaccine-induced immunity. Thus, longitudinal studies of vaccinated populations of canids are indispensable. By shedding light on these intricacies, one can address inquiries about the efficacy of the vaccine in protecting against infection.

Nevertheless, the prevailing impediments in our path pertain to the burdensome expenditures and challenges associated with conducting challenge infections. These hardships encompass the arduous task of maintaining experimental canids, the innate inability to exert control over vagrant dogs and the inherent variability observed in infection courses across diverse animal subjects [9, 92].

#### Key components of effective vaccines for canine vaccination

Key components of effective vaccines include antigens that induce adaptive immune responses, delivery systems that ensure delivery of both the antigen and the adjuvant at the right time and location, and immunopotentiators (adjuvants) that signal the innate immune system to potentiate the antigen-specific response [120].

Using next-generation vaccines represents a promising frontier in immunization, reflecting ongoing efforts to develop safer, more effective and easily deployable vaccines.

The different components involved in developing next-generation vaccines for echinococcosis in dogs are as follows.

#### Antigen selection

Next-generation vaccines for echinococcosis in dogs involve the selection of appropriate antigens derived from the *E. granulosus* tapeworm genetic sequence. These antigens should elicit a strong immune response, leading to protective immunity against the parasite. The development of multi-epitope vaccines, supported by bioinformatics to identify critical antigenic epitopes, may offer enhanced immune protection, safety and stability, outperforming traditional subunit vaccines in terms of efficacy [84, 121].

Genetic mapping has the potential to affect the development of vaccines that elicit strong immune responses against parasites and can also help overcome the hurdles posed by parasitic organisms’ genetic diversity and adaptability nature.

Through ortholog analysis, 33 ortholog groups consisting of 42 *E. granulosus* genes found solely in parasitic helminths were identified. These ortholog groups show potential as valuable targets for drugs, vaccines or diagnostics [122].

Among the 361 genes that were identified as highly expressed in adult *E. granulosus* through mRNA transcript analysis, 55 genes displayed specific expression patterns, suggesting potential involvement in adult worm development, while these genes were silenced in the oncosphere and cyst stages [122, 123].

The egM gene family, comprising egM4, egM9 and egM123, is potentially linked to the maturation of adult worms and/or the development of eggs and is highly expressed in mature adult worms, suggesting potential opportunities for the development of vaccines for dogs [124]. Worms secrete serine protease inhibitors known as serpins to counteract the impact of canine intestinal proteases. These serpins may present potential prospects for the development of vaccines [122, 125].

Dogs play a critical role in AE transmission, particularly in western China and central Asia [123, 126, 127]. Recent evidence suggests that their influence in Europe might be more substantial than previously thought. Additionally, there is potential concern regarding a wildlife cycle in the transmission of *E. granulosus*, especially in Africa [4, 123, 128]. It might be possible to develop a single vaccine that effectively targets both *E. granulosus* and *E. multilocularis* in canines. This approach would be advantageous as these two species are prevalent in many countries across the Northern Hemisphere [123]. Thus, homologous antigens with similar or identical characteristics across different species are prime candidates for vaccine development. These conserved antigens offer cross-protection against multiple species. Pairwise gene pair analysis revealed that *E. granulosus* and *E. multilocularis* share a staggering 10,018 genes that exhibit significant sequence similarity. Among these genes, an impressive 5418 genes were found to be completely identical in both species [123].

Among various parasites, 14-3-3 proteins have been extensively studied and characterized, particularly in *E. granulosus* and *E. multilocularis*. 14-3-3 family members are universal adaptors that play crucial roles in many cellular processes, such as cell cycle regulation, differentiation, growth regulation and apoptotic cell death [129]. However, their biological functions in pathogenic protists, which carry multiple 14-3-3 protein isoforms, remain mostly unexplored [130].

Among the *Echinococcus* 14-3-3 molecules mentioned earlier, the 14-3-3f isoforms U63643 (*E. multilocularis*) and AF20790 (*E. granulosus*), collectively known as E14t, have undergone extensive investigation. These proteins have been found in different stages of the parasite lifecycle. In *E. multilocularis*, they were discovered in metacestode tissue, oncospheres and protoscoleces. Similarly, in *E. granulosus*, they were also found in protoscoleces [131, 132]. Owing to its immunogenicity and capacity to initiate protective immune responses in animal models, 14-3-3 has been explored as a potential vaccine candidate [133].

Notably, while homologous antigens have some potential, creating an effective vaccine against *Echinococcus* parasites in dogs remains a complicated and active area of research.

#### Vaccine formulation

The development of next-generation vaccines aims to optimize vaccine formulations to increase their stability, efficacy and delivery. This involves the selection of suitable adjuvants and chemotactic agents along with novel vaccine delivery platforms, and additives that enhance the immune response, to improve vaccine effectiveness, and adjuvant selection should be considered with a comprehensive understanding of the immune system and antigen-triggered responses. Classic adjuvants, including alum, MF59, AS03 and AS04, are well-established commercial adjuvants widely used in vaccine development. Adjuvant delivery systems such as virus-like particles (VLPs), Toll-like receptor (TLR) agonists [monophosphoryl lipid A (MPLA) and CpG oligodeoxynucleotide (CpG ODN)], virosomes, liposomes, synthetic adjuvants (polymeric nanoparticles), immunostimulatory complexes (ISCOMs) and nucleic acid adjuvants can act as potent immune adjuvants. These systems control the release of antigens to specific sites, protect antigen bioactivity and increase antigen circulation times, helping stimulate a potent and protective adaptive immune response. It is noteworthy that the stability and toxicity of these delivery systems and the optimal dose of vaccines are significant challenges that can be addressed through tailored engineering using targeted functional groups [134].

Some of these formulation components, such as aluminum compounds, emulsions, saponins and carbomers, have already been used in licensed products, whereas others (cytokines and polyphosphazenes) are still only evaluated experimentally [135].

Several adjuvants, including Freund's complete and incomplete adjuvants, Coil A, ISCOMs and oil, have been utilized in studies investigating candidate vaccines against *Echinococcus* in dogs. Notably, the most commonly employed adjuvant in these studies was the Quil A adjuvant, which was used in six of the studies [78, 107, 109, 110, 112, 114–117, 124, 136].

Advances in adjuvant research include the design of combination systems that incorporate two or more adjuvants to increase vaccine efficacy, limit side effects and optimize dose requirements, with cost-effective and simple formulation advantages.

#### Immunization strategy

Vaccination protocols for echinococcosis in dogs need careful consideration. Factors such as vaccine administration (route of vaccine delivery) and the revaccination schedule (booster vaccinations, the number of vaccine doses needed, the interval between doses and optimal timing) are required to achieve optimal protective immunity.

#### Immunogenicity studies and field trials

Immunogenicity studies involve the evaluation of specific antibody production, immune cell activation and other relevant immunological parameters to indicate the immune response elicited by the vaccine in dogs regarding targeting *E. granulosus*. Field trials should thus be conducted to evaluate the effectiveness of the vaccine in real-world situations, typically within canine populations located in areas where echinococcosis is prevalent. Clinical trials should be designed to assess factors such as the reduction in infection rates, disease incidence, clinical outcomes, durability and any potential adverse effects associated with the vaccine and to provide crucial information on the ability of the vaccine to confer protection against specific pathogens. Field trials and immunogenicity studies can complement each other in providing comprehensive data on the performance, effectiveness and safety of next-generation vaccines for echinococcosis in dogs. Interpretable and repeatable results are crucial for the development, refinement and regulatory approval of these vaccines, ensuring their efficacy and suitability for widespread use in the field, as exemplified by EG95 in sheep.

#### Compliance and implementation

The development of next-generation vaccines should consider compliance and implementation factors such as ease of administration, vaccine storage requirements and cost-effectiveness to ensure wide-scale adoption and accessibility.

To ensure wide-scale adoption and accessibility, the design of next-generation vaccines for echinococcosis should aim for user-friendly administration methods. This can involve developing vaccines that are easily administered by dog owners or by minimally trained personnel, reducing the dependency on veterinary professionals, such as oral vaccines (e.g. a palatable form of chewable tablet). These methods can minimize the stress associated with traditional injection-based vaccination techniques and increase compliance among dog owners in administering the vaccine to their animals. According to the available evidence, the delivery methods for introducing antigens in *Echinococcus* vaccine candidates in dogs have varied. Most studies have opted for subcutaneous (SC) delivery to investigate vaccine candidates [78, 106, 109, 110, 112, 114–117, 124, 136].

In two studies, both SC and intramuscular (IM) methods were employed [106, 112]. Additionally, some studies have explored oral antigen delivery [113, 137], and in one particular study, a combination of nasal spray and oral delivery was utilized [138]. Moreover, IM delivery of a vaccine candidate has been performed in two studies [105, 107].

The design of next-generation vaccines should strive for improved storage characteristics, such as increased thermostability and/or resistance to temperature fluctuations, to overcome challenges related to cold-chain storage and transportation. The ability of vaccines to remain effective even without strict temperature control can greatly increase accessibility, especially in resource-limited settings or areas with limited refrigerator infrastructure.

Cost-effectiveness is crucial for the implementation and widespread use of canine vaccines against echinococcosis. Vaccines should be affordable and economically viable for both dog owners and veterinary professionals to ensure their adoption on a large scale.

To increase cost-effectiveness, the design of next-generation vaccines can focus on optimizing production processes, exploring more efficient manufacturing techniques and reducing production costs. Collaboration between vaccine manufacturers and governments or nonprofit organizations can facilitate bulk purchasing, affordable pricing negotiation and subsidies to make the vaccine accessible to underserved communities or regions with limited resources. Additionally, cost-effectiveness can be improved by considering the long-term benefits of canine vaccination in reducing the echinococcosis burden. Vaccinating dogs may generate substantial cost savings by preventing the transmission of diseases to both humans and livestock. This not only reduces the direct and indirect costs associated with human surgery and other treatment expenses but also mitigates the losses incurred in the livestock industry.

Overall, addressing these factors such as ease of administration, vaccine storage requirements and cost-effectiveness of next-generation vaccines for echinococcosis in dogs can ensure their broader implementation, accessibility, scalability and successful control of the disease.

Importantly, although next-generation vaccines against echinococcosis in dogs are currently under investigation, no specific vaccine is commercially available.  Table 2 lists recommendations and requirements for designing vaccine target candidates and conducting studies to provide verifiable evidence.

**Table 2:** Representation of verifiable evidence and priorities needed for proving canid immunity against *Echinococcus* after natural infection and vaccination in trials and the underlying challenges to vaccine development

Confirmation of protective immune responses in Canid following natural exposure based on empirical studies	
Development of next-generation vaccines	Reverse vaccinology and structure-based vaccine design to identify ORFs by bioinformatics based on genome sequence for application in immunization and the use of proteomics, genomics and transcriptomics Protein subunit vaccines and multi-epitope vaccines Establishing robust expression systems for production (e.g. Eukaryotic systems) Design of DNA and/or RNA constructs, plant-derived vaccines and VLP-based vaccines Evaluation of suitable delivery system and improving route of vaccine delivery (edible, ID, IP and Sub-C) and the use of appropriate adjuvants or nano-adjuvants
Well-designed immunogenicity studies with optimum protocols	Appropriate group size and statistical analysis Consideration of factors affecting immune response results: the worm burden required for providing protection, the level of infection-acquired immunity and proportion of maturing worms under different levels of acquired natural immunity, protection and/ or reinforcement of infection-naive or previously infected dogs, information about the experimental dogs, uniformity of breed or type and variability in the course of infection Evaluation of resistant to reinfection and the potentially age-related decrease in *Echinococcus* worm burden Consideration of factors affecting antifecundity effect of vaccination: evaluation of dogs’ feces prior to necropsy for showing or excluding precocious development of worms and the possibility of progression of relatively slow developing worms into fully gravid worms over subsequent days
Evaluation of homologous protein of *Echinococcus multilocularis* in *E. granulosus*,	
Considering costs and difficulties of challenge infections, i.e. maintaining experimental canids, lack of control of homeless stray dogs and variability in the course of infections between different animals	
Selection of potent candidate for field trials	
Obtaining specific, interpretable, and reputable results	
Conducting field clinical trials and confirmation of its efficacy	
Determination of longevity of vaccine protection	
Evaluation of cross-protectiveness of vaccine	
Evaluation of the impact of dog vaccination on transmission to sheep and hence the likely effects on the transmission of CE to humans	

#### Difficulties in canine vaccine development and vaccination

Vaccine against parasitic diseases require more than scientific breakthroughs, as industrial production, partnerships and financial support are crucial. Long-term commitment and sustained funding are necessary, as the timeline from discovery to finished production can take decades [139]. Industrial large-scale production with good manufacturing practice (GMP)-grade materials involves increased expenses, time and labor intensity, requiring the establishment of necessary infrastructure [140]. Clinical trials pose significant challenges, including high costs, staffing needs and resource requirements (phase II), with trials requiring longer observation periods, greater logistical resources (phase III) and tracking of adverse events in licensed products (phase IV). The success or failure of a vaccine candidate hinges on the outcome of these trials, making vaccine development difficult to attain [140].

Vaccine development requires conclusive evidence from protective experimental studies, sustainable production and market potential. If these conditions are not met, even promising vaccines may be abandoned in the initial phase [140]. For example, a schistosomiasis vaccine was abandoned because of unsustainable production and uncertain market potential [141].

Vaccine manufacturers face constraints related to investment, laboratory certification and resources [120, 140], particularly in endemic areas where equipment and alternative approaches are limited (e.g. research/development laboratories, batch scale-up for clinical trials and full-scale vaccine production) [140]. The global demand for a verified vaccine compels manufacturers to forecast and invest in production while establishing distribution channels.

Understanding the vaccine supply chain is crucial for timely decision-making, especially in low- and middle-income countries that face cost constraints, supply chain issues (e.g. long-term stability) and logistics challenges [15]. Supply chain mapping plays a vital role in understanding the current situation (e.g. capacity, demand and estimated costs) and developing strategies to overcome these challenges. By mapping supply chains, manufacturers can achieve cost savings and ultimately improve vaccine accessibility.

Additionally, general disapproval from stakeholders including animal owners and representatives of affected countries can further affect the attractiveness of the vaccine in these areas.

Vaccine procurement bodies should shift their focus from cost per dose to cost per delivery to accurately reflect the expenses associated with a vaccine, enabling increased coverage, optimized delivery and reduced waste or human error [142, 143]; notably, this requires adequate data in terms of cost-benefit analysis of interventions, logistics systems, vaccine properties, and other relevant factors.

The number of interventions required for sheep treated with EG95 is lower than that for dogs treated with PZQ; however, the sheep population is significantly larger [92]. The frequency of PZQ dosing (4–8 times per year) also has logistical difficulties, like achieving 100% coverage of dogs > 10 years old in remote rural regions [9, 81] and other endemic areas. However, registering wildlife poses challenges, especially in a short period, and achieving high canine vaccination rates in remote rural regions with logistical difficulties can be problematic [9, 81]. Since there have been no field trials for any vaccine candidates and no information about the duration of possible protection, the need for field confirmation of long-lasting immunity remains. Additionally, factors such as the undernourishment of dogs and decreasing vaccine effectiveness over time may necessitate the use of booster vaccines. Data are needed regarding the differential effects of repeated reinfections or the absence of reinfection on the protection in the definitive host as well as the need for new booster injections in larger-scale vaccination efforts. Controlling echinococcosis in wildlife, particularly in regions with high stray dog populations, is challenging, leading to the complexities involved in implementing prevention methods, including vaccination of the definitive host. However, the development and administration of a bait-delivered vaccine offer a suitable approach to disrupt the transmission cycle of the parasite in wildlife [123, 144], as recommended by the World Organization for Animal Health (WOAH) for the use of oral rabies vaccination (ORV) in dogs to complement parenteral vaccination programs in areas where dogs are an important reservoir of the rabies virus and where it is difficult to augment parenteral vaccination coverage, especially in free-roaming dogs [145–147].

### Surveillance in control measures

Most importantly, assessing the impact of control measures and justifying the ongoing expenditure of costly control interventions are not possible without proper surveillance information. Baseline data are essential for monitoring the project's progress. For this reason, implementing various surveillance approaches in livestock, sheep dogs and humans is important. Such strategies should be employed to monitor and justify the implementation of programs over a long period.

The establishment of a proper CE surveillance system for both humans and animals is needed to reduce the disease burden of CE and monitor advancements in control programs by targeting livestock and dogs.

### CE surveillance in livestock

The reliable surveillance of echinococcosis prevalence and incidence in both human and animal cases, as well as canine infection, poses several challenges. Passive surveillance of livestock echinococcosis through continuous examination of offal at each slaughterhouse is plagued by difficulties. Prevalence data alone are insufficient; they must be accompanied by information about the precise origin of the animals and their age to be useful. Livestock, such as rams or tups, bucks or billys, and castrated animals, are sent to the slaughterhouse at a young age as routine practice. Considering the slow rate of cyst growth, failing to stratify slaughterhouse-based reports (prevalence data) by age group will result in significant underreporting of echinococcosis cases. Age-stratified data, along with comparisons of similar age groups at different time points, can be highly valuable. Importantly, slaughtered animals may not necessarily originate from the area where the slaughterhouse is located, which raises concerns about the reliability of the data obtained through this method, as unrecorded animal transport can occur. Therefore, active surveillance through slaughterhouse-based studies, a vigilant reporting system with trained staff and careful supervision can provide more reliable data on the dynamics of infection [3, 9, 42, 148]. Additionally, ultrasound screening of sentinel lambs may also be considered for detecting continuing infection transmission [149]. On the other hand, a study conducted in Hejing County, Xinjiang, reported the applicability of ultrasonography screening for detecting hepatic CE in sheep flocks and monitoring control progress in a remote mountain pasture area. The findings highlighted the significant role of older sheep, particularly culled aged sheep, in the transmission of CE. Furthermore, the study revealed that administering treatment to dogs once a year did not impact the control of echinococcosis over a 7-year period from 2014 to 2021, while EG95 has not yet been implemented in this region [150].

The lack of precise diagnostics for surveillance of CE in sheep hampers the true burden, understanding of its transmission dynamics and the effectiveness of control measures. By utilizing latent class analysis (LCA) models, researchers can estimate true infection statuses, which have been applied in livestock diagnostics to evaluate a new indirect ELISA using a recombinant antigen (AgB8/2) in Río Negro Province, Argentina. This study identifies optimal ELISA cut-offs, facilitating the potential use of this assay as a surveillance tool for detecting CE at both individual sheep and flock levels, based on the epidemiological situation, thereby enhancing management strategies in affected regions [151].

### *Echinococcus* sp. surveillance in definitive hosts

The copro-antigen ELISA has emerged as the preferred screening method because of its superior balance between sensitivity and specificity, alongside its heightened suitability when contrasted with alternative approaches such as necropsy. Necropsy, a time-intensive process with stringent laboratory requirements, applies only to deceased animals. On the other hand, arecoline testing not only entails hazards but also shows reduced sensitivity. Consequently, the copro-antigen ELISA is an optimal solution in this domain [42, 148]. DNA detection in feces and environmental samples (e.g. soil, water, sewage and vegetables) has become increasingly important [123, 152–155]. LAMP-based assays and PCR are valuable tools for screening and assessing infection status under field conditions [156–158].

### CE surveillance in humans

The importance of establishing a robust and reliable surveillance system should not be overlooked, especially considering the prevalent issues of underreporting and insufficient sensitivity in official or governmental surveillance data. A comprehensive and effective monitoring system plays a pivotal role in addressing these challenges and ensuring accurate and reliable data collection. To implement control programs and evaluate their impact, it is essential to have suitable surveillance data, which can be collected quarterly in the initial stages and annually during the implementation of control programs [9]. In regions where CE prevails, the establishment of a well-suited national CE registry system that includes records of surgical and treatment data in a standardized and easily accessible format is imperative. A commendable initiative in this context is the European Register of Cystic Echinococcosis (ERCE), which has garnered participation from 44 centers spanning 15 countries. The ERCE serves as a valuable foundation, supplementing hospital-derived data to provide a comprehensive understanding of the epidemiology of clinical CE and generate evidence-based recommendations through the collection of clinical data. To overcome current challenges and accomplish its objectives, the ERCE will undergo expansion into the International Register of CE (IRCE), with a revamped framework. This expansion is aimed at addressing critical issues and bolstering the capabilities of the registry [159, 160].

To effectively evaluate the CE status in areas where it prevails, the utilization of portable ultrasound devices and hospital registry records can play a pivotal role in conducting regular and active mass screening surveys [161]. The WHO has proposed enhancing the utilization of ultrasonography as a crucial measure for eradicating echinococcosis [162] because of its portability and relatively low cost. Ultrasound is the sole imaging technique capable of assessing the prevalence of CE in remote field settings, where it can operate on solar power or small generators. Its classification capabilities facilitate stage-specific treatments. Research on the diagnosis of echinococcosis is urgently needed in hyperendemic areas.

Active mass screening via ultrasound significantly enhances the assessment of CE prevalence at the community level by including known, new and asymptomatic cases [9].

Endemic communities in remote regions, such as pastoral areas and plateaus in western China, central Asia, eastern Africa and northern and eastern Europe, primarily rely on ultrasonography for screening echinococcosis lesions because of its portability and lower cost [4, 163, 164].

This method aids in evaluating intervention effectiveness by age-specific ultrasound prevalence data, as evidenced by studies in regions like Rio Negro, Argentina [165], and allows for longitudinal studies when communities are screened regularly. However, ultrasound has limitations, particularly in detecting pulmonary cases, which necessitates using other imaging techniques such as mobile x-ray units [9].

In regions with limited livestock data, ultrasound prevalence can serve as a valuable alternative for measuring control efficacy. While serological screening has its limitations in sensitivity and specificity, ultrasound should remain the primary diagnostic tool, complemented by serology.

Magnetic resonance imaging (MRI) and computed tomography (CT) are often unavailable because of high costs, despite providing better resolution for diagnosing echinococcosis than ultrasonography [123, 163, 166].

Accurate diagnosis of echinococcosis via ultrasonography faces some main challenges: overlapping ultrasound features with other liver lesions, reliance on the radiologist's experience leading to inter-reader variability and a shortage of skilled radiologists in endemic areas [167–169]. Thus, improving the accuracy of ultrasound diagnosis presents a challenge in regions with limited medical resources and a shortage of experienced radiologists [170–172]. As a matter of fact, there is a shortage of skilled radiologists in many endemic regions, primarily because of poverty and limited medical resources as well as the distance of patients from hospitals [169].

Since there is currently no established protocol for artificial intelligence (AI)-assisted diagnosis of echinococcosis using ultrasound images, a deep convolutional neural network (DCNN) model based on ultrasonography has been proposed as an effective approach to identifying various types of echinococcosis, including AE. This model is suggested to support remote diagnosis, particularly in low-income regions [170]. To improve the diagnosis and management of echinococcosis, the development of clinical guidelines based on the WHO ultrasound classification of echinococcosis is crucial in local settings [3].

Pressing requirements for clear definitions of CE cases, an agreed-upon diagnostic algorithm, standardized diagnostic procedures and a defined target product profile for CE diagnostics are essential, as highlighted by the WHO in its 2021–2030 roadmap for NTDs [173].

### Mathematical modeling for control strategies

Regarding *Echinococcus* transmission, the integration of various factors and characteristics into mathematical models has proven to be an invaluable asset in devising highly effective control strategies. Mathematical modeling serves as a valuable instrument for simulating control strategies tailored to the unique transmission dynamics of echinococcosis at the local level. This approach provides crucial insights into the optimal timing and frequency of phased interventions, thereby maximizing the cost-effectiveness of control efforts for echinococcosis [174]. These models should encompass an array of crucial elements, such as time delays for parasite maturation, age-specific categorization of intermediate and definitive hosts, pathways of human transmission, the impact of density-dependent limiting factors, such as host demography, biodiversity of intermediate and definitive hosts, the role of immunity, influence of seasonal variation in infection pressure, incorporation of stochastic factors, such as age-differentiated host infections, *E. granulosus* life expectancy in definitive hosts, worm burden, acquisition and waning of immunity in dogs, etc., and generation of spatial/risk structure by incorporating environment-related variables [42, 99, 174–180].

The results of a compartmental model incorporating an additional compartment for the intermediate host (sheep), along with vaccination-induced immunity and other important control measures for CE (health education, deworming treatment for dogs and stray dog population management), are presented. Mathematical modeling suggests a combined strategy for mitigating the transmission of CE [181]. Implementing these optimal control measures in the long term can effectively eliminate the disease. However, the modeling study suggests prioritizing sheep vaccination and disposing of stray dogs over deworming of domestic dogs to minimize control costs. Additionally, reducing the population of other wild intermediate hosts while strengthening sheep vaccination programs and disposing of stray dogs is suggested to yield the most effective results simultaneously [32]. Many organizations and individuals promote rescue, rehabilitation and rehoming as ethical alternatives to culling of stray dogs. While the periodic culling of stray dogs through means such as bullet-based killing and anesthetic overdose has previously been a focus of municipalities, particularly in urban and peri-urban regions, substantial practical and ethical dilemmas have arisen, posing significant challenges to carrying out effective dog population management strategies. Additionally, this approach has not only failed but has also generated public opposition to the control program.

While a vaccine for dogs does not currently exist, modeling suggests that a dog vaccination strategy could reduce the prevalence of *E. granulosus*. Even a partially effective vaccine could lower the mean parasite abundance in dogs, leading to a decrease in cyst numbers over time. Although complete elimination of the parasite may not be achievable with this approach alone, increasing herd immunity to 75%, a pivotal function in the stabilization of *Echinococcus* populations, through vaccination could eventually lead to elimination, albeit over several decades. This strategy could be further enhanced by integrating sheep vaccination and improved control of sheep slaughtering practices [103].

A mathematical model examining the worldwide spread and sliding mode dynamics of echinococcosis indicated that effectively diminishing the outbreak of the disease or reducing the number of infections to an expected level (infected hosts and humans) requires more than just sufficient hospital resources and government interventions; it also necessitates the implementation of a robust threshold policy [182].

Several factors, including bioclimatic, anthropogenic and environmental factors (e.g. landscape characteristics), as well as socioeconomic and demographic characteristics, along with human and animal behavior (e.g. host-prey interactions, predator and prey animal behavior and feeding ecology), can significantly impact host density, the survival in the free stage and the dynamic distribution of *Echinococcus* due to its complex ecology [5, 183, 184]. Such factors should be considered to the fullest extent possible in modeling.

Environmental factors (e.g. landscape features [e.g. composition and configuration]) are likely linked to *Echinococcus* sp. occurrence at this spatial scale, along with climatic variables attributed to the seasonal and interannual variation in host density, survival of the free stage and dynamic distribution of *Echinococcus* [185, 186]. The significant impacts of new human-environment interactions, such as habitat loss associated with urbanization or agricultural expansion, physical barriers (e.g. population growth, immigration, establishment of new villages and towns around local rivers and construction of new dams, roads, water reservoirs and irrigation systems), remain important because the elimination of habitats and stopover areas results in increased densities of animals in the remaining areas or the formation of smaller habitats and wildlife species adaptations (synanthropic life), leading to ecological hot spots for pathogens, which can influence pathogen transmission [187–189], such as that of *E. granulosus* and *E. multilocularis* [174, 178, 190–193]. Notably, socioeconomic and demographic characteristics and human behavior (e.g. pastoralist lifestyles) are among the influential factors that interact with ecological determinants (i.e. the environment) to promote heterogeneous spatial patterns of echinococcosis [185].

The challenges faced by policymakers in controlling echinococcosis are due to its complexity, the varying environmental conditions that facilitate its spread and the limited evidence regarding the effectiveness of control strategies in different settings. Therefore, mathematical modeling has become popular for simulating control measures tailored to locally specific transmission conditions [174].

### New approaches to controlling echinococcosis: genetic manipulation

RNA interference (RNAi) technology and posttranscriptional gene silencing (PTGS) are cutting-edge tools that offer valuable insights into the function and characterization of genes involved in regeneration and development. Small interfering RNA (siRNA) molecules that have perfect homology with their target genes can be used to selectively silence specific genes for experimental and therapeutic purposes [194].

RNAi has proven effective in suppressing the expression of endogenous genes in *E. multilocularis* protoscoleces. siRNAs designed to target genes encoding 14-3-3 and ELP and delivered via electroporation induced a significant reduction in gene expression compared with that in untreated control samples [195].

A study revealed the consistent suppression of gene expression and induction of morphological changes in *E. granulosus* caused by introducing calmodulin-specific siRNA [196]. Many genes exhibit high expression levels in both adult worms and metacestodes [122]. The functional characteristics of these genes can potentially be revealed through posttranscriptional suppression via RNAi and gene knockout techniques [123].

Limited research has been conducted on *Echinococcus* regarding this matter, and the current studies are still in the early stages of exploration.

## Concluding remarks

To effectively implement targeted control programs specific to each area of endemicity, comprehensive baseline data on various epidemiological settings pertaining to both human and animal hosts can be obtained with targeted research to produce reliable and accurate information.

One crucial step toward attaining a better understanding of echinococcosis epidemiology in a given region is the establishment of a robust and reliable surveillance system and disease registry. By implementing such measures, detailed information about the prevalence, incidence, dynamics and burden of echinococcosis can be obtained. As studies on the diagnosis of echinococcosis and related subjects are urgently needed in hyperendemic areas, community-based ultrasound screenings can fill the knowledge gap regarding epidemiology and contribute to remote medical diagnosis, especially in remote endemic communities and low-income regions, where it is favored because of its portability and lower cost. Key challenges in ultrasound diagnosis include overlapping features with other liver lesions, inter-reader variability and a shortage of skilled radiologists, primarily due to poverty and limited medical resources. Addressing these challenges is essential for improving the accuracy of echinococcosis diagnosis in resource-limited settings [9, 163, 167–169].

However, improving the accuracy of ultrasound diagnosis presents a challenge in regions with limited medical resources and a shortage of experienced radiologists [170]. The development of national and international clinical guidelines based on the WHO ultrasound classification is important for improving the diagnosis and management of human echinococcosis.

Since a one-size-fits-all strategy cannot be successful, an integrated control program consisting of dog dosing with PZQ and livestock vaccination (with free-roaming dog population control when necessary) combined with health education initiatives can be practical for reducing or eliminating CE transmission in different endemic regions [3, 22], as performed in 12 years of work in Río Negro, Argentina, that is the only example of marked decrease of transmission to humans. The utilization of the EG95 vaccine to immunize intermediate hosts has been suggested as a pillar of control to effectively decrease the number of cysts accessible to definitive hosts [58].

Effective control of CE requires the promising EG95 vaccine, community engagement (strengthening stakeholder and public involvement) and alignment with socio-cultural factors under the One Health approach as achieved in South America (Argentina and Chile). Sustained dog deworming, routine vaccination of grazing animals and a robust surveillance system are essential for lasting success against this public health issue [44].

Various strategies, such as enhancing slaughterhouse facilities and practices and regulating home slaughter, as part of the overall approach, are recommended [9, 38].

Addressing dog reinfection dynamics in endemic areas is critical for formulating effective deworming strategies tailored to regional characteristics. The observed variability in reinfection patterns highlights the need for comprehensive research to fill existing data gaps on reinfection rates in endemic areas. By integrating field studies and modeling efforts, optimal deworming frequencies can be identified that balance efficacy with operational feasibility [41, 57, 81, 86]. Continuous dog deworming campaigns require enhanced coverage and the establishment of sustainable resources and infrastructure through governmental support and effective logistical enforcement, particularly in endemic regions.

Despite the absence of any existing canine vaccine, the prospect of developing a vaccine specifically designed to target dogs as the definitive host holds promise in diminishing the parasite biomass residing within the intestinal tract of dogs in endemic regions. However, within the current stage of canine vaccine development, the inconclusive nature of the findings hampers the establishment of concrete evidence supporting the existence of immunologically mediated protective responses against *E. granulosus*. The interpretability of the results and their repeatability remain subjects of discussion and should be further scrutinized in the trials. When evaluating a candidate vaccine for dogs, it is crucial to rely on multiple robust and replicated findings from well-designed studies with optimum protocols instead of relying solely on one study of a target antigen, and efforts should be made to minimize study limitations in the next studies, resulting in evaluation in potential field trials, if they have primary effectiveness. Accounting for various factors, such as those influencing immune response results and the antifecundity effect, will contribute to a better understanding and reliability of the study.

Consequently, the scientific community must continue making advancements, whereby persistent endeavors are necessary to elucidate the complexity of dog immunity. Moreover, concerted investment should be fostered, a sustained fusion encompassing the production of a promising vaccine candidate and its formulation alongside rigorous evaluation in field trials, but this endeavor presents significant challenges and requires considerable time.

## Data Availability

No datasets were generated or analysed during the current study.

## Abbreviations

CE: Cystic echinococcosis
AE: Alveolar echinococcosis
NTDs: Neglected tropical diseases
GDP: Gross domestic product
WHO: World Health Organization
DALYs: Disability-adjusted life-years
EMRO: Eastern Mediterranean Region of the WHO
PZQ: Praziquantel
CNR: Catch, neuter, and release
GPS: Geospatial technology
IoT: Internet of things
UAVs: Unmanned aerial vehicles
VLPs: Virus-like particles
TRL: Toll-like receptor
MPLA: Monophosphoryl lipid A
ODN: Oligodeoxynucleotide
ISCOMs: Immunostimulatory complexes
SC: Subcutaneous
IM: Intramuscular
GMP: Good manufacturing practice
WOAH: World Organization for Animal Health
LCA: Latent class analysis
ERCE: European Register of Cystic Echinococcosis
IRCE: International Register of Cystic Echinococcosis
AI: Artificial intelligence
DCNN: Deep convolutional neural network
RNAi: RNA interference
PTGS: Posttranscriptional gene silencing
siRNA: Small interfering RNA
